# Clinical profile and outcome of multisystem inflammatory syndrome in children (MIS-C) associated with COVID-19 infection: a single-center observational study from South India

**DOI:** 10.1186/s43054-022-00156-5

**Published:** 2023-01-23

**Authors:** Brundavanam Venkata Krishna Sai, Hema Kumar, Thirunavukkarasu Arun Babu, Raghuvamsi Chaitra, Diptirekha Satapathy, Vinoth Kumar Kalidoss

**Affiliations:** 1 Department of Pediatrics, Rainbow Children’s Hospital, Vijayawada, Andhra Pradesh India; 2 Department of Pediatrics, All India Institute of Medical Sciences (AIIMS), Mangalagiri, Andhra Pradesh India; 3 Department of Pediatric Critical Care and Pulmonary Medicine, Rainbow Children’s Hospital, Vijayawada, Andhra Pradesh India; 4 Department of Community and Family Medicine, All India Institute of Medical Sciences (AIIMS), Mangalagiri, Andhra Pradesh India

**Keywords:** COVID-19, SARS-CoV 2, Multisystem inflammatory syndrome in children (MIS-C), Children, IVIg

## Abstract

**Background:**

Multisystem inflammatory syndrome in children (MIS-C) is a post-infectious sequelae of acute COVID-19 infection affecting children. This study was done over a period of 12 months from December 2020 to November 2021 to describe the clinical presentation, laboratory abnormalities, and outcome of children with MIS-C.

**Methods:**

Seventy-eight children below 12 years of age who satisfied the WHO diagnostic criteria for MIS-C were included in the study. Clinical parameters were recorded at admission. Relevant laboratory investigations, radiological studies, and outcome were documented.

**Results:**

The most commonly affected age group was 6–12 years with a female predominance. COVID RTPCR was negative in all patients. Most cases presented 2–6 weeks after the onset of acute COVID-19 infection. Lethargy, poor feeding, vomiting, abdominal pain, loose stools, cough, and cold are common symptoms of MIS-C syndrome in children and the common signs were rash, conjunctival congestion, hypotension, tachycardia, tachypnea, and hypoxemia. Gastrointestinal system was the commonly affected followed by the hepatic, renal, and cardiovascular systems. Coronary artery abnormalities were seen in 20% of cases. IVIg was the mainstay of therapy used in 95% of patients. Mortality was 1.3%. Cases responded well to IVIg and steroids.

**Conclusion:**

Overall, the short-term outcome was favorable with low mortality in our study cohort. One-fifth of children had coronary artery abnormalities during acute phase underscoring the need for long-term follow-up.

## Background

A cluster of “pneumonia of unknown etiology” cases were reported in the Wuhan province of China in December 2019. It was later found to be caused by a highly transmissible, novel coronavirus, SARS CoV-2 which was re-designated as COVID-19 by World Health Organization (WHO). This COVID-19 infection rapidly spread to most of the countries of the world in early 2020 and was declared a pandemic by WHO in March 2020 [1, 2]. Though in the initial days of pandemic it was found that children had milder disease compared to adults, a unique, severe inflammatory disease which resembled Kawasaki disease (KD) was reported from May 2020 onwards in many children who recovered from COVID-19 infection [3–5]. Since this condition had a striking temporal association with SARS CoV-2 infection, it was later designated as multisystem inflammatory syndrome in children (MIS-C) associated with COVID-19 infection [6]. This potentially life-threatening complication of COVID-19 infection may occur during or after recovery in symptomatic as well as asymptomatic children. Spectrum of MIS-C may range from mild disease to severe involvement including shock, multiorgan dysfunction, coagulopathy, respiratory failure, myocardial dysfunction, and encephalopathy [7].

The existing treatment guidelines for MIS-C in children are extrapolated from guidelines to treat Kawasaki disease, since both these conditions have significant overlapping features [6–9]. Considering the relative paucity of data, there is an urgent need to study the clinical profile and outcomes of MIS-C based on the current treatment guidelines, so that evidence-based modifications, if required, can be done.

## Methods

This prospective observational study was done in a tertiary care teaching hospital located in South India over a period of 12 months from December 2020 to November 2021. This study was done to find out the clinical presentation, laboratory abnormalities, and outcomes of children admitted with MIS-C associated with COVID-19 infection. Written informed consent was obtained from the parents and assent from older children. The study was approved by the Institute Ethics Committee of Rainbow Children’s Hospital (No. RCHBH/188/12–2020) before the commencement of the study. All children below 12 years fulfilling the WHO diagnostic criteria for MIS-C syndrome were enrolled in the study [10]. Children suffering from chronic disorders like congenital or acquired heart diseases, asthma, bronchiectasis, chronic liver disease, chronic kidney disease, genetic disorders, IEM (inborn errors of metabolism), and neurodevelopmental disorders were excluded from the study.

After initial stabilization, detailed clinical history was obtained and documented. Clinical examination findings and results of laboratory and imaging tests were recorded. Cases were managed as per the existing guidelines for the management of MIS-C syndrome [7, 11]. Investigations to rule out common tropical infections like malaria smear and/or rapid test, dengue serology, WIDAL test, and scrub typhus serology were also performed. The treatment modalities given, response to treatment, the entire course at hospital, and outcome were documented.

### Statistical analysis

Data was collected with Microsoft Excel and analysis was done using EpiInfo Version 7.2.4. The categorical variables were summarized as frequency and percentage. The continuous variables were summarized as mean and standard deviation. The association between categorical variables was assessed using chi-square. *p*-value < 0.05 was considered statistically significant.

## Results

**Fig. 1:**
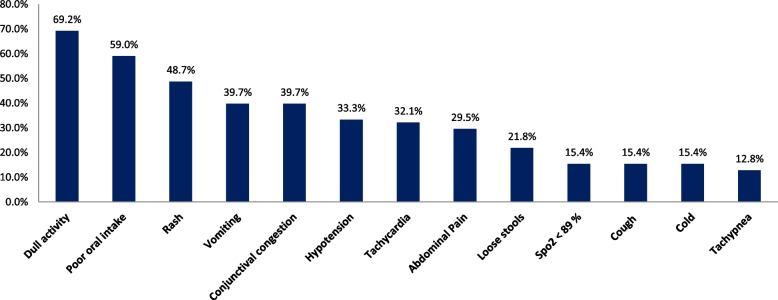
Distribution of clinical symptoms and signs among study participants

A total of 78 patients fulfilling the inclusion criteria were included in the study. The number of children less than 1 year, 1–5 years, and 6–12 years’ age group were 16 (20.5%), 28 (35.9%), and 34 (43.6%) respectively. There were 27 boys (34.6%) and 51 girls (65.4%) in our study population. Seventy-three percent of children had a definite contact history with COVID-19 infected patients. Figure 1 shows the clinical spectrum of MIS-C in the descending order of incidence among children with MIS-C. The comparison of clinical symptoms and signs in various age groups are tabulated in Table 1. The prevalence of rash (*p* = 0.03) and conjunctival congestion (*p* = 0.021) was significantly higher in older children compared to infants. The mean and standard deviation of laboratory parameters and the distribution of abnormal laboratory and radiological investigations among study participants are described in Tables 2 and 3 respectively. Leukocytosis and thrombocytopenia were commonly seen as hematological abnormalities in 44.9% and 20.5% of study population respectively. Elevated d-dimer, elevated PT-INR, and elevated aPTT were seen in 34.6%, 10.2%, and 29.5% cases respectively. The various treatment modalities received and outcome measures are described in Table 4. The proportion of children who received fluid bolus, inotropic support, renal replacement, and ventilation were 74.4%, 35.9%, 1.2%, and 7.6% respectively. Steroid and IVIg were used in 70.5% and 94.9% of study participants respectively. There was one death in our study population.

**Table 1:** Comparison of clinical symptoms and sign with demographic parameters

**Item**		**Age group**			*P*-value*
		** < 1 year** ***n* = 16 (%)**	**1–5 years** ***n* = 28 (%)**	**6–12 years** ***n* = 34 (%)**	
**Cough**		2 (12.5)	6 (21.4)	4 (11.8)	0.567
**Cold**		5 (31.3)	5 (17.9)	2 (5.9)	0.058
**Rash**		5 (31.3)	11 (39.3)	22 (54.7)	**0.030**
**Vomiting**		3 (18.8)	11 (39.3)	17 (50.0)	0.099
**Loose stool**		2 (12.5)	7 (25.0)	8 (23.5)	0.608
**Abdominal pain**		3 (18.8)	7 (25.0)	13 (38.2)	0.279
**Conjunctival congestion**		2 (12.5)	11 (39.3)	18 (52.9)	**0.021**
**Lethargy**		8 (50.0)	19 (67.9)	27 (79.4)	0.099
**Poor oral intake**		6 (37.5)	17 (60.7)	23 (67.6)	0.223
**Pulse rate**	**Normal for age**	13 (81.2)	20 (71.4)	20 (58.8)	0.234
	**Tachycardia**	3 (18.8)	8 (28.6)	14 (41.2)	
**Blood pressure**	**Normal**	12 (75.0)	21 (75.0)	19 (55.9)	0.182
	**Hypotension**	4 (25.0)	7 (25.0)	15 (44.1)	
**SpO**_**2**_	**90–100%**	11 (68.8)	26 (92.9)	29 (85.3)	0.094
	**81–89%**	3 (18.8)	1 (3.6)	5 (14.7)	
	** < 80%**	2 (12.5)	1 (3.6)	0 (0.0)	
**Respiratory rate**	**Normal**	12 (75.0)	25 (89.3)	31 (91.2)	0.244
	**Tachypnea**	4 (25.0)	3 (10.7)	3 (8.8)	

^*^Chi-square test was used. Hypotension—blood pressure < 5th centile for age; Bold p-values denote statistical significance

**Table 2:** Distribution of laboratory parameter among study participants

Investigations	Parameters (*n*)	Mean (SD)	Median	25% quartiles	75% quartiles
**COVID 19**	Antibodies titer (71)	63.9(49.8)	51.1	32.5	81.6
**Hematology**	Total leucocytes count/mm3 (71)	12,057.75 (8304.7)	10,600	6200	16,100
	Neutrophils (40)	69.1 (11.9)	71	64.25	78
	Lymphocytes (40)	25.4 (11.6)	22.5	15.5	29
	NL_Ratio (40)	3.5353 (2.1)	3.16	2.21	4.9275
	Platelet count (71)	198,450.7 (148,766.5)	160,000	114,000	230,000
**Inflammatory indicators**	CRP mg/L (71)	110.28 (96.5)	83	42	165
	ESR mm 1st hour (51)	62.76 (34.9)	60	35	88
	Ferritin ng/ml (39)	1550.23 (5861.9)	401	164	995
	LDH IU/L (12)	842.58 (1094.7)	369.5	301.5	859.25
**Coagulation profile**	D-Dimer micg/ml(35)	2.5 (2.2)	2.1	0.5	3.4
	PT (38)	16.3 (3.4)	15.4	14.175	17.175
	INR (38)	1.2 (0.3)	1.1	1	1.2
	APTT (38)	38.8 (9.8)	37.8	31.75	44
**Liver enzymes**	SGPT (61)	113.4 (429.9)	35	23	52.5
	SGOT (61)	185.3 (710.7)	52	34	69.5
**Kidney Function Test**	Urea (69)	33.2 (23.3)	26	18	43.45
	Creatinine (69)	0.7 (0.6)	0.5	0.3	0.7

*NL ratio* Neutrophil–lymphocyte ratio, *CRP* C-reactive protein, *ESR* Erythrocyte sedimentation, *LDH* Lactate dehydrogenase, *PT* Prothrombin time, *INR* International normalized ratio, *APTT* Activated partial thromboplastin time, *SGPT* Serum glutamic pyruvic transaminas, *SGOT* Serum glutamic oxaloacetic transaminase

**Table 3:** Distribution of abnormal laboratory radiological investigations among study participants

Investigations	Parameters	Frequency (*n* = 78)	Percentage
**Laboratory investigation**			
**Hematology**	Leucocytosis	35	44.9
	Elevated NLR (> 3.53)	16	20.5
	Thrombocytopenia	29	37.2
**Inflammatory indicators**	Elevated CRP (> 30 mg/l)	58	74.4
	Elevated ESR (> 40 mm/h)	38	48.7
	Elevated Ferritin (> 500 ng/mL)	17	21.8
	Elevated LDH (> 460 U/l)	4	5.1
**Coagulation profile**	Elevated D-dimer (> 0.5 µg/ml)	27	34.6
	Elevated INR (> 1.2)	8	10.2
	Elevated APTT (> 35 s)	23	29.5
**Liver enzymes**	SGPT (> 40 IU/l)	27	34.6
	SGOT (> 40 IU/l)	40	51.3
**Kidney Function Test**	Urea (> 40 mg/dl)	19	24.4
	Creatinine (> 1.1 mg/dl)	7	8.9
**Radiological investigation**			
**Chest X-ray**	Pleural effusion	4	5.1
	Pneumonia	2	2.5
**Echocardiogram**	Ventricular dysfunction	6	7.7
	Coronary artery abnormalities	15	19.2
	Small patent ductus arteriosus	5	6.4
	Pulmonary arterial hypertension	2	2.5
**Ultrasound abdomen**	Polyserositis	2	2.5
	Ascites	1	1.2
	Epididymitis	1	1.2
	Hemoperitonerum	1	1.2
	Mesenteric lymphadenopathy	1	1.2

*NL ratio* Neutrophil–lymphocyte ratio, *CRP* C-reactive protein, *ESR* Erythrocyte sedimentation rate, *LDH* Lactate dehydrogenase, *PT* Prothrombin time, *INR* International normalized ratio, *APTT* Activated partial thromboplastin time, *SGPT* Serum glutamic pyruvic transaminase, *SGOT* Serum glutamic oxaloacetic transaminase

**Table 4:** Distribution of treatment modalities and outcome of study participants

Parameters	Frequency (*n* = 78)	Percentage
**Respiratory support**		
Low-flow nasal oxygen (*LFO*)	1	1.2
High-flow nasal cannula (*HFNC*)	4	5.1
Non-invasive ventilation (NIV)	4	5.1
Mechanical ventilation	2	2.5
**Treatment**		
Fluid bolus	58	74.4
Steroids	55	70.5
IVIg	74	94.9
Aspirin	49	62.8
Inotropic support	28	35.9
Renal support	1	1.2
**Outcome**		
Death	1	1.2
Hospital stay (mean ± SD days)	4.7 ± 1.8	
ICU stay (mean ± SD days)	2.8 ± 1.5	

*ICU* Intensive care unit

## Discussion

To the best of our knowledge, this study is reporting one of the largest prospective case series of MISC syndrome published yet from India with a total of 78 cases. Our knowledge about the epidemiology, pathogenesis, clinical spectrum, and associated laboratory abnormalities seen in MISC syndrome is still evolving [7]. The diagnostic criteria of MISC are constantly revised with time as more evidence is being generated [7, 10].

Our study identified that the most commonly affected age group was 6 to 12 years accounting for more than 50% of the study population. This finding was in agreement with other similar published studies [9, 12–14]. We also noticed that girls were affected more than boys (M: F ratio of 1:1.9). Similar finding was also seen in another study by Dhanalakshmi et al. ( M:F ratio of 1:1.4 out of 19 cases) whereas most other studies showed male predisposition—Hoste et al. (M:F—1.4:1 out of 928 cases), Goldfred et al. (M:F—1.2:1 out of 570 cases), Sethy et al. (M:F—3.2:1 out of 21 cases), Kaushik et al. (M:F—1.6:1 out of 33 cases), and Sugunan et al. (M:F—1.9:1 out of 32 cases) [9, 12–16].

Though COVID RT-PCR was done in all patients, none were found to be positive. Rapid antigen test was positive in only one (1.3%) child. COVID-19 antibody was positive in 69 (88%) children. Fifty-four (69%) children had a history of contact with COVID positive cases. Other studies have shown RT-PCR positive in 19% to 31% of cases [9, 13, 15]. In our population, we noted a 2–6-week lag period for MIS-C presentation following COVID-19 infection or contact with COVID-19 case.

The most common presentations were lethargy (69%), poor oral intake (59%), rash (48%), vomiting (39.7%) and conjunctival congestion (39.7%). The presence of rash and conjunctival congestion were proportionately higher with the increasing age of children. Gastrointestinal system involvement was seen in 62% of our study population which was lower than global data of 86% as reported by recent systematic reviews [14, 17].

Leukocytosis, high N/L ratio and thrombocytopenia were found in 45%, 20%, and 37% of patients respectively. C-Reactive protein (CRP) was elevated in 58 (74%) patients. 38 (48%) had elevated ESR [18]. Serum Ferritin, d-dimer, and LDH were elevated in 21%, 34%, and 5% respectively. In contrast, elevated CRP, ferritin, and d-dimer were found in a higher proportion of children in the reported literature [17].

Fifty-four percent of patients had biochemical evidence of hepatic dysfunction as evidenced by elevated SGOT, SGPT, or both, similar to a study published from South India [9]. In our study, 25.7% of patients had renal dysfunction as evidenced by elevated serum urea or creatinine or both and 1 (1.3%) required hemodialysis. In a study by Williams et al., incidence of AKI was 35% and 2% required renal replacement therapy [17]. Epididymitis was seen in only 1 study subject. In a multi-center cohort study, 1 out of 232 children of MIS-C developed epididymitis [19].

Hypoxemia was observed in 15% of patients at presentation out of which 4% had SpO2 below 80%. Eight percent of patients had abnormal chest radiograph. Eleven (14%) children required respiratory support and 2 (2.6%) children needed invasive mechanical ventilation. The need for invasive mechanical ventilation was ranging from 0 to 39% in published studies [8, 9, 15].

Nine percent of our patients had serositis (5.4% had pleural effusion, 1.4% had ascites, and 2.7% had polyserositis). One systematic review revealed the incidence of pericardial effusion to be 22% [14].

Thirty-three percent of children were hypotensive at presentation. In one study, the incidence of hypotension at admission was found to be 47% [13]. Cardiac involvement was found in a significant number of cases in our population. The most common abnormality was coronary artery dilation which was observed in 19.2% of cases. Another study revealed the similar incidence of coronary artery involvement [15]. Incidence of myocardial systolic dysfunction was relatively less (7.7%) in our study compared to 63% in data from New York by Kaushik et al. [16]. Fifty-eight (74.3%) of children presented with shock out of which 28 (35.9%) were fluid refractory requiring inotropes. Similar results were reported by Hoste et al. [14]. In studies from New York by Kaushik et al., 51% required vasopressor support [16].

While neurological involvement was seen in up to 48% of cases in some studies, our population had only 5.1% of children presenting with seizures and altered sensorium [13].

Though IVIg, steroids, anticoagulation, and aspirin are the mainstay of therapy in MISC syndrome, there is limited evidence to support their use. Due to the striking similarities of this syndrome with Kawasaki disease, the same treatment is being recommended for MIS-C as well [7, 14, 17]. Our study group received IVIg as the first-line treatment followed by steroids, anticoagulation, and aspirin but we did not use biological agents. Seventy percent of our patients received both IVIg and steroids. There is no evidence-based consensus for managing MIS-C syndrome which reinforces the immediate need for such studies.

The overall prognosis was good in our patients when compared to global data. The mean duration of hospital stay was 4.7 days. And mean duration of ICU stay was 2.8 days.

Out of 78 children, 77 survived and 1 child (1.3%) died. Most of the other Indian and western studies also revealed similar incidence of mortality, ranging from 0 to 3%. Only one study from Odisha reported 9% mortality [13].

The main limitation of this study is the small sample size and lack of follow-up. Long-term follow-up can throw more light on chronic complications of MIS-C in children.

## Conclusion

Lethargy, poor feeding, vomiting, abdominal pain, loose stools, cough, and cold are common symptoms of MIS-C syndrome in children and the common signs include rash, conjunctival congestion, hypotension, tachycardia, tachypnea, and hypoxemia. The incidence of rash and conjunctival congestion increased significantly with the increasing age of the child. The gastrointestinal system was the most commonly affected system in MIS-C followed by the hepatic and cardiovascular systems. Cases responded well to IVIg and steroids. Overall, the short-term prognosis was good in our study population. Largescale multi-centric data on MIS-C is required to understand this novel complication of COVID-19 infection in children.

## Data Availability

The datasets used and/or analyzed during the current study are available from the corresponding author on reasonable request.
